# I Was Merely a Brick in the Game: A Qualitative Study on Registered Nurses' Reasons for Quitting Their Jobs in Hospitals

**DOI:** 10.1155/2024/6662802

**Published:** 2024-03-06

**Authors:** Josefin Bäckström, Ulrika Pöder, Ann-Christin Karlsson

**Affiliations:** ^1^Department of Health, Education and Technology, Nursing and Medical Technology, Luleå University of Technology, SE-971 87 Luleå, Sweden; ^2^Department of Medical Sciences, Psychiatry, Uppsala University, SE-751 85 Uppsala, Sweden; ^3^Department of Public Health and Caring Sciences, Caring Sciences, Uppsala University, SE-751 22 Uppsala, Sweden

## Abstract

The aim was to explore why registered nurses (RNs) in Sweden choose to quit their jobs in hospitals, also in relation to experienced patient safety. Previous research has shown that nurse turnover, especially in hospital settings, is a serious challenge for society and health care globally. Insufficient staffing of RNs is linked to poorer patient outcomes and a general patient safety at risk. It is, therefore, important to continually explore how nurses describe their reasons for quitting their jobs. The study was conducted using a qualitative descriptive design, based on 11 semistructured interviews with RNs. The analysis generated four categories describing the results: Feeling that the profession is not valued; Psychological and physical symptoms related to work; An insufficient and unsupportive organization; and Unsatisfying leadership and teamwork. Specifically, the RNs participating in this study described a range of reasons for quitting, where the feeling of not being valued and treated as a respected and autonomous profession was a common thread throughout the results. RNs experienced that, overall, the insufficient work conditions, also resulting in lower patient safety, ultimately led to their decision to quit. The findings highlight the crucial need for employers to develop working conditions for RNs, to make sure that the profession is valued according to professional standards and provide the potential for autonomous nursing practice. To reduce nurse turnover, and instead attract and retain nurses, leadership and management in nursing need to be adjusted to meet the demands of a modern academic profession.

## 1. Introduction

From a global perspective, nurse turnover, which can be defined as employees choosing to leave an organization or workplace, has been a serious challenge for the healthcare sector for decades [1–3]. The healthcare sector has for long been pinpointed as struggling to recruit and retain nurses, even more so in the aftermath of the COVID-19 pandemic [4]. In Sweden, the national situation in acute care hospitals has recently been described by national authorities as alarming and unacceptable regarding patient safety, primarily due to the employers' inability to recruit and retain nurses [5].

The causes of nurse turnover have been studied extensively over the years. Vital aspects that might influence nurses' decision to remain in their workplace are aspects related to leadership and management, career options, organizational environment, and number of colleagues [6, 7]. Additionally, generational differences can play a role [6, 8]. Several of the causes identified in the studies as reasons for why nurses quit, for example, poor work environment, are deemed preventable [9].

Job satisfaction and a healthy work environment are central factors that have an impact on nurses' turnover intentions [10–14]. Qualitative studies have identified a link between a well-functioning work environment and the quality of patient care involving nurses and nurse midwives [15, 16]. Quantitative research exhibits variations in the definitions of work environment and the source of patient safety ratings, whether derived from nurses or nursing indicators, studies consistently highlight a connection between nurses' work environments and a range of nursing outcomes. These encompass, but are not restricted to, missed care, post-discharge mortality risk, incidents of falls, medication errors, and occurrences of pressure ulcers [14, 17–19]. One central aspect of high-quality care is patient safety, which by definition is fundamental to delivering health care that is effective, safe, and person-centered, and also prevents and reduces risks [20]. The consequences of nurse turnover are not limited to serious economic values but also comprise the effect on staffing and on nurse—as well as patient outcomes [2, 21].

Despite a substantial amount of scientific research on the topic of nurse turnover, myths continue to flourish on why nurses choose to quit, risking meaningless or even contra-productive interventions by employers striving to turn the trend. Previous research has emphasized the need for further studies in this area to focus on equity and the wellbeing of nurses, and on the underlying mechanisms of nurse turnover [21, 22]. Moreover, it is suggested that effective strategies to enhance retention and raise healthcare standards may exhibit variability among hospitals. Accordingly, these intricacies should be considered when devising and implementing interventions targeted at improving nurse retention [6], where qualitative studies are posited to contribute significantly to a more profound understanding of the nuanced and underlying factors influencing nurse turnover. To our knowledge, few qualitative studies have focused on how registered nurses (RNs) in a retrospective perspective describe job satisfaction and patient safety in relation to having quit their jobs, e.g., nurse turnover. Therefore, in this study, we aimed to explore why RNs in Sweden choose to quit their jobs in hospitals, also in relation to patient safety.

## 2. Materials and Methods

### 2.1. Design

The study was conducted using a descriptive design with a qualitative approach.

### 2.2. Informants and Setting

Informants were purposefully recruited through a university hospital with approx. 900 beds, and one county hospital with approx. 160 beds, in two healthcare regions in Sweden. A list of 53 RNs, who had quit their jobs in either medical or surgical units during 2018, was obtained through the respective HR departments. Only RNs on permanent contracts were included. An information letter, including an informed consent form, was sent by regular mail to all the potential informants. The first author made contact by telephone to follow-up on any questions, and if the contacted person was interested in participating, a time for an interview was scheduled. Eleven RNs finally participated. The recruitment resulted in variation regarding age (range 26–63), gender (men and women participated), and experience as RNs (range 4–34 years since graduation). Four informants held a postgraduate diploma in specialist nursing, with or without an additional one-year master's degree. One participant held a Ph.D.

### 2.3. Interviews and Procedure

Data were collected from March 2020 to February 2021, using a semistructured interview guide. At the beginning of the interview, the informants were asked to describe some background characteristics. Below are examples of the main questions in the interview.

- How would you describe your job satisfaction as a RN in your former workplace?
- Can you describe why you experienced/did not experience job satisfaction?
- Could you describe what would have been important for you to feel job satisfaction?
- Some describe a relation between job satisfaction and patient safety; what are your thoughts about this–considering your former workplace?
- What are the reasons that you chose to quit your former job?
- What would make you rethink and return to your former workplace?

Interviews were carried out online or by phone, were audio recorded, and transcribed verbatim including emotional expressions. The interviews ranged between 32 and 62 min.

### 2.4. Data Analysis

The interviews were analyzed using systematic text condensation inspired by the description by Malterud [23]. The text was read by the authors to identify the preliminary themes. Text units that were considered relevant to the aim were identified and sorted according to these themes. Then, code groups were generated by the first author. Thereafter, all authors were involved in the reformulating and reconstructing of the codes until a consensus was reached. The content in each code group was re-contextualized and, subsequently, synthesized into four categories, describing the results. Illustrative quotes were used to strengthen the credibility of the results.

### 2.5. Ethical Considerations

All informants had signed a written informed consent form and were asked if anything was unclear before the interview began. All were informed that participation was voluntary and that they could withdraw from participation at any time without an explanation or questions being asked. Informants were told that data would be handled with confidentiality and that data would be presented without exposing any of the informants in a way that could cause them to be identified. None of the informants withdrew from the study. Ethical approval was obtained from the Regional Ethical Review Board (Reg. no. 2016/111).

## 3. Results

The analysis resulted in four categories which describe the registered nurses' reasons for quitting their jobs in the hospitals, including aspects of patient safety, where it influenced the decision.

### 3.1. Feeling That the Profession Is Not Valued

The RNs experienced that the employer did not value formal competence, such as a higher academic degree and senior expertise, and that visible career options are lacking. They expressed a wish to develop professionally, but the only career path that was evident was becoming a ward manager, also described as the only way to achieve an increase in salary. The informants felt that the salary was too low in relation to their formal competence and experience, which was an important factor influencing their decision to quit.

RNs felt that their professional development had stagnated, and an increase in salary was seen as impossible to negotiate outside the annual salary evaluation. Informants felt that they were seen as a collective rather than individual professionals, and that individual skills and competencies were not valued. RNs expressed that the employer encompassed a dated view on their competencies, such as the typical skills related to academic degrees in general. Career positions and roles for nurses where the individual's competence was valued were experienced as rare. Academic career paths and roles were not evident, which they expressed should be apparent already upon entering the organization.

*Easiest for managers that all [nurses] are the same, just go in and do their job and nothing more. Do not ask any questions or show any personal interest. […] It is perceived as a problem in healthcare that nurses should preferably be streamlined. Everyone should be equal, be equally experienced, everyone should do the same.* (Informant 10)

*[…] it's as much about tradition and culture. So that's … it's going to take a very long time to adjust or change. But I think it's about changing one's view of what is the ultimate goal for a nurse simply. […] Because that's the thing with, should we be seen as a bit, like just a name on the schedule, or should we be seen as a competent [professional], so. And that is, of course, you must not close your eyes to the fact that we all need to fill a gap for care to work, of course. We need to make sure that there are nurses in place, but then you need to create a tradition and culture that sees the importance of our core competence.* (Informant 8)

### 3.2. Psychological and Physical Symptoms Related to Work

The overwhelming work situation with extreme stress was described as causing both physical and psychological symptoms, leading to an unbearable situation. Being physically tired but still not being able to relax, constant stress, and having a stomach in knots, the feeling that “I am going to die here,” not being able to think, not being able to concentrate, experiencing cold sweat, a racing heart, and the feeling of “enough is enough” – all contributed to making the decision to quit. The stress caused RNs to feel their head spinning with thoughts after getting home from work and made it difficult for them to regain energy. Knowing that the stress caused dangerous situations for the patients added to the feeling of unsatisfying work situation. The stress and the overall work environment were described as a risk factor for missed care, threatening patient safety.

*[…] I couldn't think, I didn't know what… I didn't know the time, I could concentrate on … or I couldn't sleep, because I was working three shifts.* (Informant 5)

*And if I want to be at work then, maybe I am happier, have more energy and have more motivation to work, which then … My attitude toward my work affects the patients, because they see me at my job. If I'm angry, sad or happy, it probably shows in me and how I behave toward the patients. And if I then feel unmotivated and depressed or feel “oh God, what a job… I don't even want to be here,” I might miss certain things that my patient says, for example, just because I think that … or in case I have my thoughts in another place, etcetera. So, there are so many different factors that come into play. So, I definitely think that if you are satisfied, there will be increased patient safety. I think, it will definitely be.* (Informant 1)

### 3.3. An Insufficient and Unsupportive Organization

RNs felt that an organization in the workplace supporting nursing was lacking, making it tough to perform their duties according to the professional nursing standards. The working environment was described as important, both to ensure patient safety and as an overall important aspect of work satisfaction. Permanent and extensive understaffing, especially regarding RNs, was viewed as having a substantial impact on the decision to quit. Due to the extreme work overload, sometimes the informants could not see the patients they were supposed to care for during a whole shift and had to discuss patients during rounds that they had not even met themselves. RNs were aware that patients needed them and that they were very ill–not being able to attend to their needs caused stress and made them feel they were not doing a professional job. They had difficulties in finding the time to even go to the bathroom themselves. Having to supervise students continually, and at the same time feeling new in the profession, added to the general burden and stress. Feeling bad toward colleagues was experienced as disheartening. Newly recruited colleagues could leave the workplace within months, and some even chose to leave the profession within a year.

*[…] if you think about patient safety in the care unit, it means that resources must be available so that you can do your job and have time to see the patients. I feel that, it's really the most important thing, organizationally speaking. That it improves … yes, you need to have staffing and you need to have time, so that you have time to see your patients. Among the worst shifts is where you don't have time to see patients.* (Informant 12)

Situations when the RNs had to care for patients who were outsourced to other wards than the one intended were described as especially stressful, since they did not feel they possessed the adequate competence and experience regarding the specific condition for which the patient was getting treatment.

*There were a lot of shifts where […] you found your nursing colleagues in the medicine room crying, and I never thought I'd be a part of that. But I was involved.* (Informant 1)

Informants described that the working hours and scheduling had played an important role in the decision to quit. Work schedules were described as lacking flexibility, and the employer was unwilling to consider individual needs when organizing the schedule. Especially working days, evenings, and nights in a mix–sometimes resulting in only a few hours of sleep before the next shift–was deemed especially draining. The inflexible schedules were viewed as something that could cause illness and made it impossible to have a social life outside of work and to manage family obligations.

*[…] it was this shift, above all, that it was so very messy with day, evening, day, evening and then every other weekend. So that was probably the big thing.* (Informant 14)

*[…] so it's development opportunities, it's the salary issue, it's the working environment. Then I would say … part of this, which I have admittedly been a little bit into, but which I have reflected on a lot, is connected to this thing of working three shifts and doing a rotation. But it's really about flexibility, in terms of working hours. Because it is also the case that people find themselves in different situations in one's life during one's life cycle.* (Informant 8)

### 3.4. Unsatisfying Leadership and Teamwork

The RNs described leadership as a crucial factor influencing their decision to quit. This was described in terms of collegial support from both senior colleagues and from managers. Informants felt abandoned when being the only one that others turned to for advice and support, while they had no support from senior colleagues themselves; instead, they were solely dependent on the hospital physician on-call. Managers did not offer support, and nursing assistants were sometimes the only ones in the team with a longer experience. This was described as unsatisfying, since assistants lack knowledge of the scope of practice for RNs. Clinical supervision in nursing and senior support from colleagues with a higher qualification was described as necessary, but nurse practitioners and RNs with specialist competence were viewed as being too busy to provide adequate support. Informants also acknowledged that managers find themselves in unbearable situations where they are aware of the shortcomings and the poor work environment. Managers were also described as absent, not taking responsibility, and blaming each other–problems that were addressed were met with no response.

*I think there is a lack of support and guidance in that case. Because I think that regardless of whether you are in a very critical or stressful situation, and you have colleagues next to you who can support you, I think that you can feel satisfied after a work shift. But if you don't have that, I think there is a lot that is missing. Because several times, I even thought, “no, but this is not the profession I should have anymore.”* (Informant 1)

Nursing management was described as insufficient and lacking leadership that would have been required to create an attractive work environment. Managers were experienced as absent and lacking interest in patient safety and the situation of the employees. Informants experienced that managers did not adequately address the individual professionals' interests, neglecting to value competencies and promote lifelong learning. Negative feedback and a lack of positive feedback were deemed discouraging. RNs described that managers were often new in their positions and were changed frequently. Managers' formal qualifications were questioned when they expressed a lack of interest in academic merits–something that informants could experience as a reason for quitting after they had accomplished a higher degree themselves.

*They may not be disinterested, but it is probably overshadowed by the fact that they have other problems, organizational problems. That the department should be staffed 24 hours, with the right professions, that … yes. I don't think they are completely disinterested, but they are not schooled in it either, since there were two non-academic nurses [managers]at the time. Or one was academic but had no interest in academia …* (Informant 10)

*And, of course, I can also turn to my assistant nurses. We had a lot of people who were experienced and had worked there for ten, fifteen years. But they are not familiar with this particular … in our profession and what we do; so, it felt like I was talking to a wall sometimes. And when you tried to contact the manager about these problems, it wasn't often that they were there either. Or they were in meetings, were busy with something completely different, and didn't have time. So, it was probably a big part of why I finally felt that “enough is enough.”* (Informant 1)

RNs also described that a hierarchical structure in the workplace, lacking team collaboration and including interprofessional relations, contributed to their decision to leave. Physicians could be unfriendly and unsupportive; also, other RNs could act hostile, for example, when a colleague had achieved a new formal qualification, such as a higher academic degree. The overall atmosphere could be negative and unsupportive. Physicians could demand to be formally titled, and RNs could be ignored when greeting them. Rude behavior from physicians toward RNs was experienced as diminishing, and nursing as a profession was viewed as having an overall low status in the organization. Male RNs were deemed to be less exposed to hostile behavior than female RNs. Moreover, informants thought that a lack of informal socializing between physicians and RNs had a negative impact on the teamwork. They also experienced an overall inequality between professions in the hospital, namely, that physicians have a clearer career path than they themselves after professional achievements, including academic degrees.

*I had a situation when I was fairly new and working nights, where I was scolded for even daring to ring the back-up on call doctor instead of the primary on call doctor. But I thought, he didn't take me seriously. And when you called the back-up on call doctor, there was still someone who listened to you straight away. And then we could still help the patient, but then you were scolded for doing it that way.* (Informant 4)

## 4. Discussion

The results in this study comprise four categories: Feeling that the profession is not valued, Psychological and physical symptoms related to work, An insufficient and unsupportive organization, and Unsatisfying leadership and teamwork. RNs who have chosen to leave their jobs as registered nurses in an acute care hospital describe a range of reasons for doing so. There were various reasons including feeling overwhelmed, consequences related to stress; a lack of a patient safety culture; an overarching experience of being treated unfairly and not according to what would be expected based on qualifications; not having the prerequisites required to practice their profession; poor leadership; and a lack of a nursing organization and insufficient career options, together with dysfunctional interprofessional teamwork.

The results show that the RNs' work situation with experiences of extreme stress caused both psychological and physical symptoms, experienced as linked to threatened patient safety. This is consistent with previous research regarding RNs' experiences of round-the-clock care, and how constant stress might have a serious impact on health and well-being, underpinning the decision to leave the workplace [16], with the feeling that “enough is enough.” RNs felt the stress, along with a lack of recovery and replenishment of new energy between work shifts, was a risk factor for missed nursing care, thus experienced as threatening patient safety. This missed nursing care, also described as care left undone [24, 25] or unfinished nursing care [26], has been associated with lower patient safety [19, 27]. Missed nursing care, apart from the consequences for the patients, is associated with absenteeism, job dissatisfaction [28–32], poor retention and staff morale, turnover intention of nursing staff [26], but most of all, a burden on patient safety [19, 33]. Identifying current and up-to-date research in line with the findings in this study, unsurprisingly, did not present a challenge. This can be seen as noteworthy, since the topic has been addressed for decades, pointing out basically the same challenges and consequences for both patient safety and nursing as a profession [34, 35]. This highlights the utmost importance that the, now, substantial scientific evidence on factors affecting work environment and nurse turnover [10–14] should seriously be considered in national and international policy, and by decision-makers in healthcare at all levels.

A connecting thread running through the categories was that the RNs in this study did not experience that they were treated as a respected *profession* by their employer, but rather as a replaceable workforce with a modest professional value to the organization. This shed further light on aspects of how RNs can be understood in large organizations, such as hospitals, and underlying aspects of what needs to be taken into account in reforming such organizations in order to facilitate recruitment and retention of RNs.

Employers were described as comprising an outdated view on RNs, not corresponding to what would be expected regarding a profession. Few visible career options, besides positions in ward management, were offered; moreover, employers did not seem to value higher academic degrees. Expectations on professional development were not met; furthermore, the working conditions, including salaries, did not make the RN's feel valued. These results are congruent with previous research in Swedish contexts, indicating that employers might not seek out autonomous academically trained professionals when recruiting RNs, but rather a workforce that is primarily expected to act as independent medical assistants, valued for their practical skills and dedication [36]. The medical perspective has been dominant in healthcare for centuries; it is only in the past decades that it has been challenged by other more holistic approaches to health [37], such as nursing, which originates from a holistic, caring, and person-centered approach to health [38]. Perhaps partly explained by being a “new profession” [39], RNs have struggled to gain influence and power, both in health policy as well as in hospital organizations. Nursing is still commonly seen as subordinate to medicine by the society [40]. This perception is also reflected by the results in our study, describing unequal career options compared to physicians and unsatisfactory interprofessional teamwork, where RNs might experience derogatory behavior from physicians. *If* the profession is still seen as subordinate to physicians, both in the team and at the organizational level, it might come as no surprise that RNs experience treatment as disparaging, and that such experiences in the long-term contribute to their decision to quit.

Somewhat surprisingly, the RNs in this study did not specifically bring up the need for an overarching structure for nursing leadership, although deficient and incompetent management at the ward level was emphasized as a reason for quitting. The explanation for this remains unclear, given that the specific question on overarching organizational leadership structures was not addressed in the interviews. Rodríguez-Pérez et al. [40] have articulated a need for nurses to strengthen their influence and leadership by gaining positions in faculty, professional organizations, and in health policy. Leadership, in general, is a central reason for nurse turnover, described in this study as well as in a substantial number of previous research studies [6, 7]. Regardless of the fact that healthcare organizations are constantly changing, thus affecting the healthcare staff [41, 42], it is unclear to what extent the professionalization of nursing [39] has had an impact on organization, and employers' core views on nursing as a profession. The introduction of new public management [43] in healthcare has also been pointed out as reducing professional governance and influence in general, conceivably making it even more difficult for new professions to strengthen their mandates in such organizations. In continuously slimming organizations such as acute hospitals, traditionally run by the dominating medical perspective [37], it is conceivable that nurses have not reflected upon, or been able to influence the organization enough to create a career structure, allowing the profession to grow and develop substantially, also beyond bedside nursing.

As pointed out in previous research [44], organizational change in healthcare is best received when staff experience the need and value for change, especially regarding better patient care. The experiences shared by the RNs in this study indicate that the organizations they worked in had not fully integrated the status and value of them as full professionals. Not all hospitals in Sweden, for example, have positions as Chief Nursing Officer/Chief Nurse Executive in the highest organizational level, neither a clear structure nor a formal regulation for nursing leadership that runs from “top to bottom.” Organizational culture and quality of leadership, have been linked to the ability to recruit and retain nurses in hospitals [6]. The results of this study underscore that RNs need to take actions to ensure that the profession gains mandates required to develop the organizational structures, where skills and education can thrive and are valued.

The academic progression and academic degrees in nursing, constituting a fundament in claiming professional status, are evidently linked to better patient outcomes and lower mortality [45, 46]. The results in this study indicate that, regardless of the development of nursing in Sweden during the past decades, originally initiated by the Swedish Higher Education Reform of 1977 [47] where nursing was integrated into regular university education, the parallel development in the employers' views might not have occurred. Nurses claim to fill the criteria of a profession [39], but other professions and organizational structures might not agree with this –which might be a somewhat overlooked cause when it comes to research on nurse turnover [7, 9, 16]. The results in this study suggest that it is important that the profession shoulder the ongoing process to address the need for structural change in both academia and healthcare. This is needed to ensure that RNs not only see possibilities of a professional career in healthcare but are also allowed and expected to practice nursing at its highest standards. A healthcare organization, which allows nurses to provide safe and person-centered care in a structure, where RNs are expected to lead the profession in well-functioning interprofessional collaborations on all levels, likely increases the chances of both recruiting and retaining nurses for the long term.

## 5. Methodological Considerations

The interviews in this study were conducted during the early outbreaks of the COVID-19 pandemic. This might have had an impact on the possibility of recruiting informants due to the overall, extreme burden in healthcare at the time. Nevertheless, the informants in this study focused on a work situation that made them quit their jobs, which took place before the pandemic. Therefore, we believe that the results are not impacted by the extraordinary situation during the pandemic. Moreover, there is a potential for recall bias given the two-year gap between the termination of employment and the interviews. However, this time lapse might have allowed the informants to share experiences that were more reflective and less influenced by immediate and intense emotions surrounding the decision to quit. Nevertheless, despite this temporal delay, the interviews were characterized by richness and detail.

A facilitating factor in recruitment was that interviews were conducted online through phone or digital meetings. Both men and women participated in the study; however, as expected, due to the gender distribution in nursing in general, there were fewer men than women. The authors, therefore, chose not to specify the exact numbers to protect the integrity of the informants. Trustworthiness was achieved by carefully describing the context, and by a transparent and reflective communication between the authors in the whole process. This study was carried out by three women, all registered nurses. All worked as senior lecturers, and two also worked part-time with nursing quality development projects in hospitals. The authors have broad experience of conducting research interviews, and from the qualitative analysis method applied in this study. Transferability to other settings seems possible, but it is up to the reader to decide [48]. The authors had no previous relationship with the informants, and the study followed the Standards for Reporting Qualitative Research (SRQR) [49].

## 6. Conclusion

The RNs in this study expressed various reasons leading them to the decision to quit their jobs. These experiences ranged from overwhelming feelings of lacking the practical and organizational prerequisites to practice nursing at a professional standard, to severe understaffing together with insufficient scheduling and low salaries. Additionally, the RNs expressed the feelings of being valued and treated as a subordinate profession, not being expected to have the same expectations on professional autonomy and career possibilities as other professions. The RNs experienced that the, overall, insufficient work conditions, including an experienced lack of patient safety in several dimensions, ultimately pushed them to quit their jobs. Hence, RNs expressed a wish and expectation to practice their profession according to professional standards, and to be treated like a self-governing profession in the team and organization – but ultimately, they realized they were only seen by the employer as a replaceable brick in the game.

### 6.1. Implications for Nursing Management

The findings underscore the vital necessity for nursing management to develop overall working conditions and a leadership structure for RNs to ensure that the profession feels valued according to the professional standards and requirements for autonomous practice. Nursing management and leadership need to be adjusted to meet the expected demands of a modern academic profession and move away from a potential collective view of RNs. To reduce nurse turnover and, instead, attract and retain RNs, the organizations ought to encompass a view on RNs as individual professionals, who are expecting the same treatment and career options as traditional academically trained professions. Nevertheless, it is equally important to underscore that the pervasive changes required to alter the perception of nursing as a profession cannot be accomplished solely by individual nurses or nurse managers, regardless of their level. Such changes also need to be promoted and implemented at the political and societal level, both locally and internationally. Since the challenges of retaining nurses are not confined to local or national contexts but are observed internationally, it is crucial to consider the specific situation in each country, which may influence how these issues should be addressed.

## Data Availability

The interview data used in this study are available in the Swedish language upon reasonable request from the first author.
